# Prevalence of preclinical Alzheimer disease: Comparison of current classification systems

**DOI:** 10.1212/WNL.0000000000005476

**Published:** 2018-05-08

**Authors:** Silke Kern, Henrik Zetterberg, Jürgen Kern, Anna Zettergren, Margda Waern, Kina Höglund, Ulf Andreasson, Hanna Wetterberg, Anne Börjesson-Hanson, Kaj Blennow, Ingmar Skoog

**Affiliations:** From the Department of Neuropsychiatric Epidemiology Unit (S.K., J.K., A.Z., M.W., H.W., A.B.-H., I.S.) and Clinical Neurochemistry Laboratory (S.K., H.Z., K.H., U.A., K.B.,), Department of Psychiatry and Neurochemistry, Institute of Neuroscience and Physiology, Sahlgrenska Academy at the University of Gothenburg, Sweden; and UCL Institute of Neurology (H.Z.), Queen Square, London, UK.

## Abstract

**Objective:**

To determine the prevalence of preclinical Alzheimer disease (AD) according to current classification systems by examining CSF from a representative general population sample of 70-year-olds from Gothenburg, Sweden.

**Method:**

The sample was derived from the population-based H70 Gothenburg Birth Cohort Studies in Gothenburg, Sweden. The participants (n = 322, age 70 years) underwent comprehensive neuropsychiatric, cognitive, and somatic examinations. CSF levels of β-amyloid (Aβ)_42_, Aβ_40_, total tau, and phosphorylated tau were measured. Preclinical AD was classified according to criteria of the A/T/N system, Dubois 2016, National Institute on Aging–Alzheimer's Association (NIA-AA) criteria, and International Working Group-2 (IWG-2) criteria. Individuals with Clinical Dementia Rating score >0 were excluded, leaving 259 cognitively unimpaired individuals.

**Results:**

The prevalence of amyloid pathology was 22.8%, of total tau pathology was 33.2%, and of phosphorylated tau pathology was 6.9%. With the A/T/N system, the prevalence of A+/T−/N− was 13.1%, A+/T−/N+ was 7.3%, A+/T+/N+ was 2.3%, A−/T−/N+ was 18.9%, and A−/T+/N+ was 4.6%. When the Dubois criteria were applied, the prevalence of asymptomatic at risk for AD was 36.7% and of preclinical AD was 9.7%. With the NIA-AA criteria, the prevalence of stage 1 was 13.1% and stage 2 was 9.7%. With the IWG-2 criteria, the prevalence of asymptomatic at risk for AD was 9.7%. The *APOE* ε4 allele was associated with several of the categories. Men more often had total tau pathology, A+/T−/N+, preclinical AD according to Dubois 2016, asymptomatic at risk for AD according to the IWG-2 criteria, and NIA-AA stage 2.

**Conclusion:**

The prevalence of pathologic AD markers was very common (46%) in a representative population sample of 70-year-olds. The clinical implications of these findings need to be scrutinized further in longitudinal studies.

The hallmarks of Alzheimer disease (AD) include the aggregation of β-amyloid (Aβ) into plaques, the hyperphosphorylation of tau protein with the formation of tangles, and brain atrophy.^1^ In neuropathologic series, a large proportion of cognitively normal elderly have Alzheimer pathology.^2^ In the Cognitive Function and Ageing (CFAS) population study, one-fifth of elderly without dementia fulfilled neuropathologic criteria for AD, and one-third had moderate to severe plaque pathology.^3^ The corresponding figure in those with dementia was 59%.^3^ PET studies report amyloid pathology in ≈30% of healthy elderly from control or convenience samples.^4–7^ CSF studies show frequencies ranging from 12% to 36%.^8–11^

Most data are derived from convenience samples, e.g., normal controls from memory clinics or volunteers. Data from representative samples are needed to clarify the population prevalence of preclinical AD pathology.

Biomarkers reflecting the accumulation of Aβ deposition are the earliest sign of AD in healthy elderly.^12,13^ Aβ pathology is detected earlier in CSF than in PET.^12,14^ Brain autopsy and biomarker studies indicate that amyloid pathology is initiated ≈10 to 20 years before clinical symptoms.^15^ Presently, there are 4 different classifications in use for preclinical AD. The most recent, the A/T/N system, was introduced in 2016.^16^ The consensus group–defined criteria for preclinical AD (Dubois criteria)^17^ came in 2016, the International Working Group-2 (IWG-2) criteria^7^ in 2014, and the National Institute on Aging–Alzheimer’s Association (NIA-AA) criteria^18,19^ in 2011. The only classification that differentiates between phosphorylated (p)-tau and total (t)-tau is the A/T/N system. The prevalence of preclinical AD in accordance with these different classification systems needs to be elucidated in representative population samples. The aim of the current study was to determine the prevalence of preclinical AD in accordance with the 4 current classification systems based on CSF data from a representative population-based sample of 70-year-olds.

## Methods

The baseline sample was derived from the 2014 to 2016 examinations of the H70 Gothenburg Birth Cohort Studies in Gothenburg, Sweden. The sample was obtained from the Swedish Population Registry and included persons living in private households and individuals in residential care.^20^

Every 70-year-old in Gothenburg, Sweden, born during 1944 on prespecified birthdates was invited to the examination in 2014 to 2016, and 1,203 participated (response rate 72.2%). Of these, 430 (35.8%) consented to a lumbar puncture (LP). Contraindications (anticoagulant therapy, immune-modulated therapy, cancer therapy) were present in 108, leaving 322 (26.8%). CSF volume was insufficient for 4 participants.

For the purpose of the present study, we defined our study cohort as cognitively unimpaired as operationalized by a Clinical Dementia Rating (CDR) score of 0. Thus, participants with CDR score >0 (n = 63) were excluded, leaving 259 participants with a CDR score of 0.

### Standard protocol approvals, registrations, and patient consents

All participants and/or their close relatives gave written informed consent. The study was approved by the Regional Ethics Review Board in Gothenburg.

### Assessments

Participants were examined at the neuropsychiatric memory clinic at Sahlgrenska University Hospital in Gothenburg or at home. Experienced psychiatric research nurses performed the neuropsychiatric examinations, which comprised ratings of psychiatric symptoms and signs, and tests of mental functioning, including assessments of episodic memory (short-term, long-term memory), aphasia, apraxia, agnosia, executive functioning, and personality changes.^21–23^ Key informant interviews were performed by psychiatric research nurses as described previously.^21^

Examinations included the Mini-Mental State Examination (MMSE) and the CDR. A geriatric psychiatrist and neurologist (S.K.) assigned the final ratings.

Dementia was diagnosed according to the DSM-III-R criteria^21^ because these criteria have been used in the Gothenburg studies for >30 years.

Stroke and TIA information was acquired from self-reports and key informants. The participants underwent comprehensive somatic examinations.^22^ Education, defined in years of education, was assessed by self-report or close informant information.

### *APOE* genotyping

The single nucleotide polymorphisms (SNPs) rs7412 and rs429358 in *APOE* (gene map locus 19q13.2) were genotyped, with a success rate of 100%, with the KASPar PCR SNP genotyping system (LGC Genomics, Hoddesdon, Herts, UK). Genotype data for these 2 SNPs were used to define the ε2, ε3, and ε4 alleles.

### CSF sampling and biomarker analyses

LPs to collect CSF samples were performed in the L3-4 or L4-5 interspace in the morning.^24^ The first 10 mL CSF was collected in a polypropylene tube and immediately transported to the laboratory for centrifugation at 1,800*g* at 20°C for 10 minutes. The supernatant was gently mixed to avoid possible gradient effects, divided into aliquots in the polypropylene tubes, and stored at −70°C.^10,24^

CSF total tau (t-tau) and tau phosphorylated at threonine 181 (p-tau) were determined with a sandwich enzyme-linked immunosorbent assay (INNOTEST htau Ag and PHOSPHO_TAU [181P], Fujirebio [formerly Innogenetics], Ghent, Belgium).^25,26^ CSF Aβ_42_ was measured with a sandwich enzyme-linked immunosorbent assay (INNOTEST Aβ_1–42_) specifically constructed to measure Aβ starting at amino acid 1 and ending at amino acid 42.^27^ For the Aβ_42_/Aβ_40_ ratio, the V-PLEX Aβ Peptide Panel 1 (6E10) Kit (Meso Scale Discovery, Rockville, MD) was used.^28^ All assays are included in the panel of clinical routine analyses at the Mölndal Clinical Neurochemistry laboratory. Analytic runs had to pass quality control criteria for the calibrators, and internal quality control samples had to be approved. The CSF cutoffs in this study were as follows: CSF Aβ_42_ levels ≤530 pg/mL, CSF t-tau levels ≥350 pg/mL, and p-tau levels ≥80 pg/mL.^10^

### A/T/N classification

According to the A/T/N classification scheme,^16^ each participant was classified into 3 binary categories. A refers to Aβ pathology (CSF Aβ_42_ levels ≤530 pg/mL), T to pathologic p-tau (CSF p-tau ≥80 pg/mL), and N to neurodegeneration biomarker (CSF t-tau ≥350 pg/mL). Participants can have 8 possible biomarker combinations.

### The Dubois 2016 criteria

In accordance with the criteria from Dubois et al.,^17^ persons with both amyloid and tau pathology were classified as having preclinical AD. Cognitively normal participants (i.e., CDR score 0) with either amyloid pathology or tau pathology are considered to be at risk for AD (AR-AD).

### IWG-2 criteria

According to the IWG-2 criteria,^7^ asymptomatic at risk for AD is defined as cognitively normal persons (i.e., CDR score 0) with Alzheimer pathology (here defined as CSF Aβ_42_ levels ≤530 pg/mL) and pathologic CSF t-tau or p-tau (CSF t-tau levels ≥350 pg/mL or p-tau levels ≥80 pg/mL). We were not able to use the IWG-2 criteria of increased retention of fibrillary amyloid PET.

### NIA-AA criteria

The NIA-AA criteria for preclinical AD include 3 stages. Stage 1 refers to asymptomatic individuals with abnormal amyloid markers; stage 2 refers to asymptomatic individuals with abnormal amyloid and injury markers (markers of neurodegeneration such as high CSF t-tau or p-tau, neuronal dysfunction on fluorodeoxyglucose-PET, cortical thinning, and hippocampal atrophy on MRI); and stage 3 refers to individuals with subtle cognitive changes and abnormal amyloid and injury markers.^18^ An additional category, suspected non-AD pathophysiology (SNAP), defined as abnormal tauopathy without amyloidopathy, was later suggested.^19^ In our study, only stage 1, stage 2, and SNAP were included; all were based on CSF injury markers only.

### Overlap between current classifications systems

The following categories coincide here:

1. A+/T−/N−, A−/T−/N+ or A−/T+/N−, or A−/T+/N+; AR-AD according to Dubois 2016; and the combination of SNAP (isolated tauopathy) and stage 1 (isolated amyloidopathy) in the NIA-AA criteria.
2. A+/T+/N−, A+/T−/N+, or A+/T+/N+ according to A/T/N; preclinical AD in the Dubois criteria; stage 2 of the NIA-AA criteria; and asymptomatic at risk for AD according to the IWG-2 criteria.
3. A+/T−/N− and stage 1 according to the NIA-AA criteria.
4. Total pathology of Dubois 2016 criteria and the NIA-AA criteria.

### Statistical methods

Differences in proportions and means were tested with the Fisher exact test and *t* test.

To address the concern that the prevalence figure could be biased because only 36% consented to an LP, we used propensity score weighting. First, we conducted a binary regression model in those with CDR score of 0 in the total sample to identify variables that predicted participation in CSF. We considered male sex, education, depression according to DSM-V, self-reported stroke, age, systolic blood pressure, living alone, and income. In a final model, we used male sex, education, depression according to DSM-V, and self-reported stroke. From this, a probability of accepting the CSF was calculated for each person, and the inverse of this probability defined the weights to be used in a weighted calculation of the sample prevalence. The weighted prevalences were very similar to the unweighted (see Results). Because these differences were regarded as trivial, we chose to use unweighted prevalences for the sake of clarity.

A 2-tailed level of significance was used (*p* < 0.05). Statistical analyses were completed with SPSS for Windows (version 17; SPSS, Chicago, IL), SAS (version 9.4; SAS Institute Inc, Cary, NC), or STATA (version 14; StataCorp, College Station, TX).

### Data availability statement

The authors state that anonymized data on which the article is based will be shared by request from any qualified investigator.

## Results

Table 1 gives the baseline characteristics of participants and nonparticipants in the CSF examination. The dementia prevalence for the entire sample was 2.3% (n = 28).

**Table 1:**
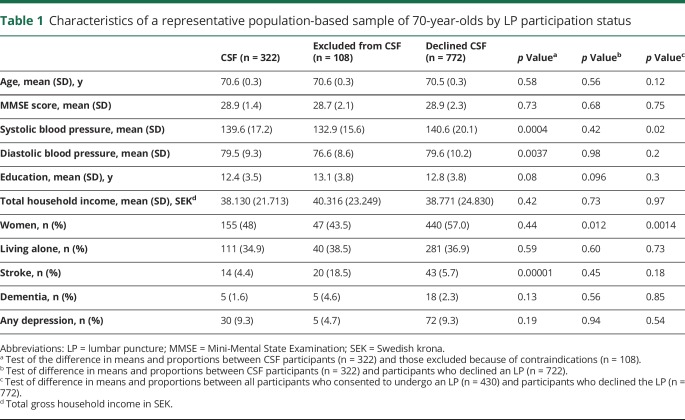
Characteristics of a representative population-based sample of 70-year-olds by LP participation status

Those who were excluded from the LP because of contraindications (n = 108) were similar regarding age, sex, MMSE score, living alone, years of education, economic status, and prevalence of dementia and depression, but they had more often had strokes and had lower mean systolic and diastolic blood pressures than participants with CSF data (table 1). Among those with CSF, 259 (80.4%) had a CDR score of 0, 60 (18.6%) had a CDR score of 0.5, and 3 (0.009%) had a CDR score of 1. Participants with CSF were similar to the rest of the sample regarding age, MMSE score, mean systolic and diastolic blood pressures, education, economic status, living alone, and prevalence of stroke, dementia, and depression, but they were more often male (table 1). The coefficient of variation for the different quality control samples used for each biomarker assay was in the interval of 3.6% to 9.9% (table e-1, links.lww.com/WNL/A424). The CSF biomarker levels, ranges, and variations are given in table e-2. The distributions for Aβ_42_, t-tau, and p-tau were slightly skewed; therefore, the mean and median were provided. Aβ_40_ was normally distributed. The distributions for Aβ_42_, t-tau, and p-tau were similar to those seen in Alzheimer's Disease Neuroimaging Initiative ^29^ (table e-2).

Despite the small number, those with dementia (n = 5) had a lower mean level of Aβ_42_ ( 428.2 vs 718.9 ng/L, *p* = 0.004), lower Aβ_40_ level (4,781.8 vs 6,220.3 pg/mL, *p* = 0.02), lower Aβ_42_/Aβ_40_ ratio (0.55 vs 0.87), higher t-tau level (531.2 vs 331.1 ng/L, *p* = 0.001), and higher p-tau level (67.6 vs 49.4 ng/L, *p* = 0.021) than those without dementia (table e-2, links.lww.com/WNL/A424).

Among those without dementia, there was no difference between those with a CDR score of 0.5 (n = 57) and those with a CDR score of 0 (n = 259) in Aβ_42_ (697.4 vs 724.5 ng/L, *p* = 0.41), Aβ_40_ (6,052.7 vs 6,250.9 pg/mL, *p* = 0.31), Aβ_42_/Aβ_40_ ratio (0.86 vs 0.88, *p* = 0.53) t-tau (328.3 vs 332.0 ng/L, *p* = 0.83), and p-tau (49.1 vs 49.5 ng/L, *p* = 0.86) levels.

The analyses that follow include only those with a CDR score of 0 because a CDR score of 0.5 is an exclusion criterion in all classifications.

### Prevalence of amyloid and tau pathology

Among those with a CDR score of 0, the prevalence of amyloid pathology was 22.8%, of t-tau pathology was 33.2%, and of p-tau pathology was 6.9% (table 2). The weighted prevalence of amyloid pathology was 22.5%, of t-tau pathology was 33.7%, and of p-tau pathology was 7.1%. Because these differences must be regarded as trivial, we chose to use unweighted prevalences in the rest of the results for the sake of clarity.

**Table 2:**
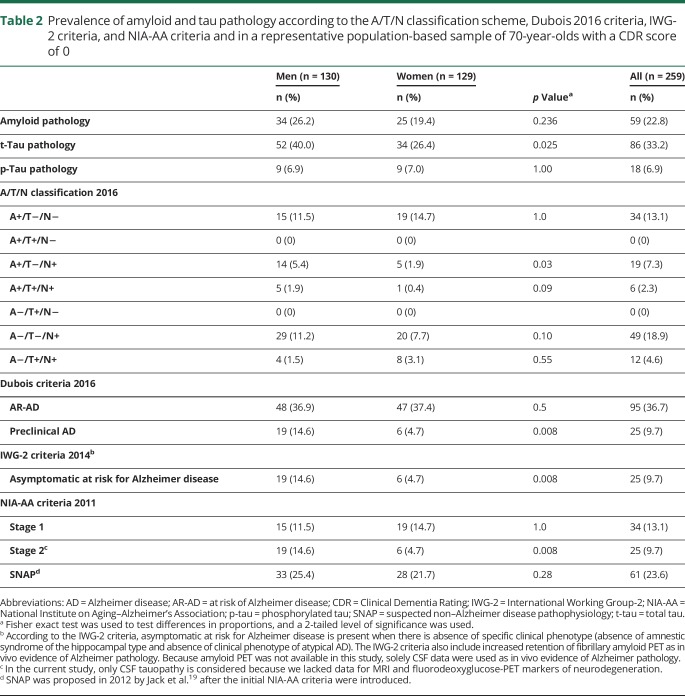
Prevalence of amyloid and tau pathology according to the A/T/N classification scheme, Dubois 2016 criteria, IWG-2 criteria, and NIA-AA criteria and in a representative population-based sample of 70-year-olds with a CDR score of 0

### A/T/N system

The prevalence of A+/T−/N− was 13.1%, of A+/T−/N+ was 7.3%, of A+/T+/N+ was 2.3%, of A−/T−/N+ was 18.9%, and of A−/T+/N+ was 4.6%. No participants had the biomarker combination A+/T+/N− and A−/T+/N− (table 2 and a Venn diagram in the figure).

**Figure:**
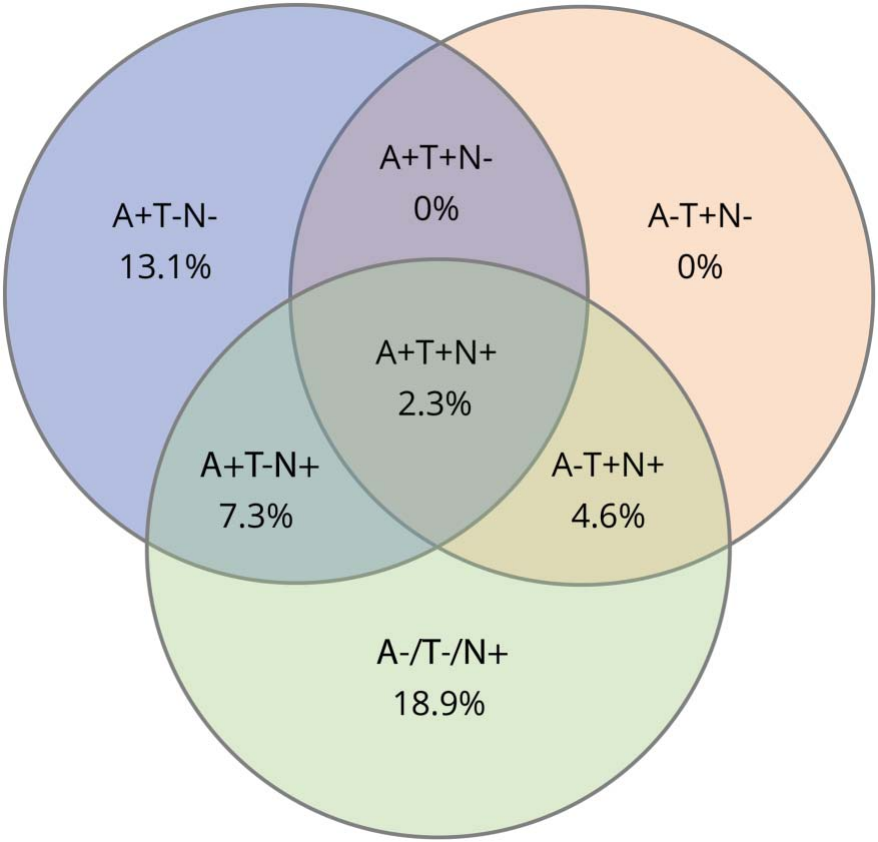
Venn diagram Venn diagram of the ATN distribution of amyloid and tau pathology according to the A/T/N classification scheme in a representative population-based sample of 70-year-olds with a Clinical Dementia Rating score of 0. A+ refers to Aβ pathology (CSF Aβ_42_ levels ≤530 pg/mL), T+ to pathologic p-tau (CSF p-tau ≥ 80 pg/mL), and N+ to neurodegeneration biomarker (CSF total tau ≥350 pg/mL) in 259 cognitively normal elderly all 70 years of age. Aβ = β-amyloid; p-tau = phosphorylated tau.

### Dubois 2016 criteria

The prevalence of AR-AD was 36.7% and the prevalence of preclinical AD 9.7%. Thus, in total, 46.4% had AR-AD or preclinical AD (table 2).

### IWG-2 criteria

The prevalence of asymptomatic at risk for AD according to the IWG-2 criteria was 9.7% (table 2).

### NIA-AA criteria

The prevalence of NIA-AA stage 1 was 13.1%, of stage 2 was 9.7%, and of SNAP was 23.6% (table 2).

The CSF biomarker levels, ranges, and variations in the different classification systems are given in table e-3, links.lww.com/WNL/A424.

### *APOE* ε4

Among persons with a CDR score of 0, *APOE* ε4 allele carrier (n = 86) had lower Aβ_42_ (606.4 vs 785.3 ng/L, *p* ≤ 0.0001), lower Aβ_42_/Aβ_40_ ratio (0.76 vs 0.94, *p* < 0.00001), higher t-tau (375.5 vs 311.0 ng/L, *p* = 0.0005), and higher p-tau (54.6 vs 47.1 ng/L, *p* = 0.0016) levels compared to *APOE* ε4 noncarriers (n = 168) (table e-4, links.lww.com/WNL/A424). There was no difference in Aβ_40_ levels (6,319.1 vs 6,237.9 pg/mL, *p* = 0.89). All participants with pathologic values for all 3 biomarkers (n = 8) were *APOE* ε4 carriers. Among participants with dementia (n = 5), 4 had the *APOE* ε4 allele (*p* = 0.017).

The *APOE* ε4 allele was more common in participants with A+/T−/N−, A+/T−/N+, and A+/T+/N+; AR-AD and preclinical AD according to Dubois 2016 criteria; stage1 and stage 2 according to NIA-AA criteria; and asymptomatic at risk for Alzheimer disease according to the IWG-criteria (tables 3–5).

**Table 3:**
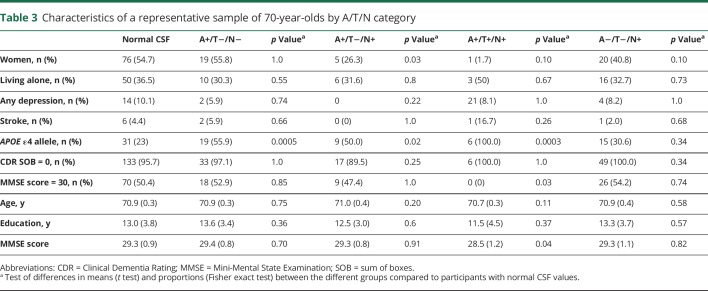
Characteristics of a representative sample of 70-year-olds by A/T/N category

**Table 4:**
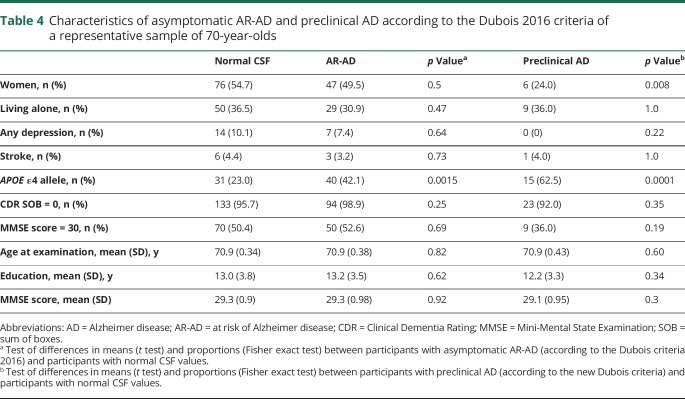
Characteristics of asymptomatic AR-AD and preclinical AD according to the Dubois 2016 criteria of a representative sample of 70-year-olds

**Table 5:**
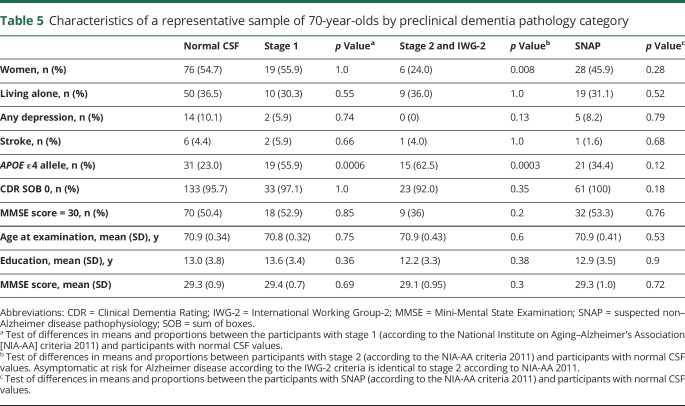
Characteristics of a representative sample of 70-year-olds by preclinical dementia pathology category

### Other characteristics

Men more often than women had t-tau pathology A+/T−/N+, preclinical AD according to Dubois 2016, asymptomatic at risk for Alzheimer disease according to the IWG-2 criteria, and NIA-AA stage 2 (tables 3–5). Participants with A+/T+/N+ had a lower mean MMSE score (28.5 vs 29.3, *p* = 0.04) than other participants. There were no differences between different categories and participants with normal CSF values regarding age, living alone, prevalence of depression, stroke, and years of education (tables 3–5).

### Analyses of 70-year-olds with good cognition

Finally, we examined the subgroup scoring 30 on the MMSE. The prevalence of the different categories of preclinical AD was similar in this group compared to the rest of the sample with a CDR score of 0 (tables 3–5). None of the participants with A+/T+/N+ had an MMSE score of 30.

## Discussion

We applied CSF data from a population study to determine the prevalence of preclinical dementia and related conditions in accordance with 4 commonly used classification systems. AD pathology was observed in almost one-half of the 70-year-olds with a CDR score of 0. Almost one-fourth had amyloid pathology and a third had tau pathology, representing neuronal injury. Our findings show that amyloid and tau pathology on CSF is very common in cognitively normal populations, as previously shown in neuropathologic series and in convenience samples using PET.^2,30^

Regarding the A/T/N system, we report findings nearly identical to those in a recent report from the Mayo Clinic on persons 50 to 95 years of age for A−/T+/N+ (4% vs 4.6% in our study), A+/T−/N− (10% vs 13.1% in our study), and A+/T−/N+ (8% vs 7.3% in our study).^31^ The lower prevalence of A+/T+/N+ in the current study probably reflects our population-based design. We did not find any cases of A+/T+/N− and A−/T+/N−, suggesting that pathologic CSF p-tau is highly correlated to pathologic CSF t-tau in a population sample of cognitively intact older people. In addition, it is of interest that we found some cases with A−/T+/N+ (4.6%) because T+ is not supposed to occur in the absence of abnormal brain amyloidosis. However, this may reflect primary age-related tauopathy,^31,32^ in which neurofibrillary tangles in brains without amyloid (Aβ) plaques are indistinguishable from neurofibrillary tangles in those with AD. It is also noteworthy that we found more cases with t-tau pathology than with p-tau pathology. These findings may reflect a combination of primary age-related tauopathy and other non-AD pathologies such as vascular disease or Lewy bodies.

With the use of the Dubois 2016 criteria, slightly more than a third were classified as asymptomatic AR-AD, and another 1/10th had preclinical AD. Regarding the NIA-AA criteria, another study, in cognitively normal volunteers (mean age 66 years),^8^ reported that the combined prevalence for stage 1 and SNAP (same as Dubois AR-AD) was 31%, a figure slightly lower than in our study (36.7%). That study also reported that 12.3% had isolated amyloid pathology (stage 1), a figure almost identical to that in our study (13.1%). The proportion of participants with CSF amyloid or tau pathology in accordance with IWG-2 criteria (46%) in our study parallels findings based on Alzheimer's Disease Neuroimaging Initiative data.^33^

We found that 23% had CSF amyloid pathology. This figure is similar to those reported from neuropathologic series of older people^2^ and from convenience samples using PET scans in which 20% to 30% are Pittsburgh compound B PET positive.^30,34^ Using CSF, 1 Swedish study, conducted in cognitively healthy elderly volunteers (mean age 72 years) recruited through advertisement, reported that 27% had Aβ_1–42_ levels below the study cutoff,^11^ thus paralleling our findings. Our figure is also similar (27%) to a US study of volunteers.^9^ The concordance between our findings and those from convenience samples is striking, despite the fact that our sample is representative and selected only on the basis of birthdates, while convenience samples are selected on the basis of, for example, advertisements, relatives of memory clinic patients, volunteers, or veterans.

Our study is based solely on data for persons 70 years of age. A higher figure would be anticipated in older age groups because there is strong evidence that amyloid pathology increases with age.^30,35^ A meta-analysis reported a gradual increase with age in the frequency of amyloid pathology based on PET and CSF data from 10% at 50 years of age to 44% at 90 years of age in cognitively normal participants.^35^

We observed associations between *APOE* ε4 carriership and all 3 biomarkers, as well as with the criteria of A+/T−/N−, A+/T−/N+, and A+/T+/N+. A recent study using the A/T/N system showed that participants with A+ were twice as often *APOE* ε4 carriers than participants without A+.^31^ In line with some previous studies, carriage of *APOE* ε4 was related to AR-AD and preclinical AD according to Dubois, as well as stage 1 and stage 2 according to NIA-AA and asymptomatic at risk for Alzheimer disease according to IWG-2 criteria. One study using the NIA-AA criteria found that the proportion of preclinical AD (NIA-AA stage 1–3) was higher in *APOE* ε4 carriers compared to noncarriers.^9^ Another study found that *APOE* ε4 carriers more often had preclinical AD.^8^ In a study on cognitively normal persons in the age span of 30 to 90 years, *APOE* ε4 carriers more often had amyloid positivity on PET after 70 years of age.^36^

We also found some sex differences. Pathological t-tau, A+/T−/N+, preclinical AD according to Dubois, asymptomatic at risk for Alzheimer disease according to IWG-2, and NIA-AA stage 2 were more common in men. Another study reported no sex differences with the A/T/N system, although it noted a trend for a higher proportion of men in the A−/T−/N+ group and the greatest proportion of women in the A+/T−/N− group.^31^ Men may have accumulated more nonspecific brain pathology as reflected in t-tau levels as a result of lifestyle choices such as alcohol abuse, head trauma, or vascular risk factors. The higher prevalence of preclinical AD in men among 70-year-olds is also noteworthy because later in life, after 85 to 90 years of age, clinical AD is more common in women. It could be that men with preclinical AD do not survive to the clinical stages, thus leading to higher prevalence of clinical AD in surviving women beyond 85 years of age. These questions can be answered only by longitudinal follow-up. Sex differences in preclinical AD need to be studied further.

MMSE score did not differ between those with and those without preclinical AD as identified by the different AD pathology classification systems, with the exception of the A+/T+/N+ group. Others report lower MMSE scores in preclinical AD.^9^ This disparity may be related in part to the population-based nature of our study. The cases of AR-AD and preclinical AD, stage 1, stage 2, and SNAP identified in our study likely represent an early stage in the disease process. This is further supported by the high mean MMSE scores (≈29) and the observation that the prevalence figures were similar in those with an MMSE score of 30. The fact that we found lower MMSE levels only in participants with pathologic levels of all 3 biomarkers suggests that these are probably closer to conversion to mild cognitive impairment (MCI). We could not show this for amyloid positivity and signs of neurodegeneration alone, indicating that tauopathy plays a significant role in the conversion to MCI. Further support for this comes from a study that found that the progression rate to a CDR score ≥0.5 was 5% for stage 1 and 46% for stage 2+, again showing that the combined pathology of amyloidopathy and neuronal injury is driving the conversion.^8^

The conversion rate to MCI and dementia in asymptomatic individuals with amyloid and tau pathology is still unclear. Given the overall low dementia incidence in septuagenarians,^37^ it is likely that the majority with amyloid or tau pathology in our study will not develop dementia during the coming decade. However, even if development of dementia is rare in this age group, cognitively healthy individuals with amyloid pathology on PET or CSF decline faster in cognitive function.^38^

Among the strengths of this study are the representative population-based sample, the relatively high response rate for LP, and the comprehensive examinations conducted by trained psychiatric nurses. Some limitations need to be addressed. Even if the number with CSF data in this study was relatively large, the overall number is relatively low, yielding low statistical power (i.e., subsamples). More than one-third consented to LP, but almost one-quarter was excluded because of contraindications, illustrating the challenges of conducting population-based CSF research. Although participants with CSF data were similar to the rest of the sample regarding several factors, it is possible that participants were healthier, thus creating selection bias and not true prevalence figures. However, weighted prevalence figures using propensity score modeling were very similar to the unweighted prevalence. We have most likely underestimated the true prevalence of pathology. Moreover, it is possible that there have been subtle cognitive differences between the biomarker groups that were not detected with our brief and unspecific cognitive tests. A further limitation is that we did not use PET scans. However, the correlation between CSF Aβ_42_ and PET has been shown to be high,^14^ while markers of neurodegeneration are less concordant.^8^ Finally, this is a population-based study examining Swedish 70-year-olds; therefore, results cannot be generalized to other groups.

## Glossary

Aβ: β-amyloid
AD: Alzheimer disease
AR-AD: at risk for Alzheimer disease
CDR: Clinical Dementia Rating
CFAS: Cognitive Function and Ageing
DSM-III-R: *Diagnostic and Statistical Manual of Mental Disorders, 3rd edition, revised*
DSM-V: *Diagnostic and Statistical Manual of Mental Disorders, 5th edition*
IWG-2: International Working Group-2
LP: lumbar puncture
MCI: mild cognitive impairment
MMSE: Mini-Mental State Examination
NIA-AA: National Institute on Aging–Alzheimer’s Association
p-tau: phosphorylated tau
SNAP: non–Alzheimer disease pathophysiology
SNP: single nucleotide polymorphism
t-tau: total tau
